# Outcomes of critical patients with tracheostomy: proposal of a decannulation protocol

**DOI:** 10.1186/2197-425X-3-S1-A933

**Published:** 2015-10-01

**Authors:** C Duro, C Granja, S Castro, A Cardoso, S Mestre

**Affiliations:** Hospital of Faro, Emergency and Intensive Care Department, Faro, Portugal; University of Algarve, Department of Biomedical Sciences and Medicine, Faro, Portugal

## Introduction

Tracheostomy (TCT) places an high social and economical burden not only for patients and their families, but also for institutions and the management of their beds. Longitudinal studies are contradictory, due to different non-standard practices, different circuits of these patients in the hospitals and the differentiation of human resources. Therefore, the creation of decannulation protocols can be of major importance.

## Objectives

1) To describe patients who underwent TCT in the Intensive Care Unit (ICU) and analyse their outcomes at 28 days and 6 months;

2) To determine if there is a relationship between the location of decannulation and the outcomes;

3) To propose a decannulation protocol.

## Methods

Retrospective analysis of data collected in ICU of Faro Hospital, during 1 year and 3 months. Patients who underwent upper thoracic surgery and/or neck surgery, previous TCT and had decision to do not resuscitate, were excluded (n = 33). The following clinical data were collected: type of TCT (percutaneous vs surgical); reason for TCT; reason for ICU admission; age; hospital and ICU length of stay ; duration of mechanical ventilation (MV); early vs late percutaneous TCT duration; place of discharged. Follow up at 28 days and 6 months; failed attempts to decannulation; place of decannulation, readmission; and severity of disease at ICU admission. Chi-square tests for categorical variables, non-parametric Kruskal-Wallis and independent samples Student t test for continuous variables, were performed.

## Results

**Figure 1:**
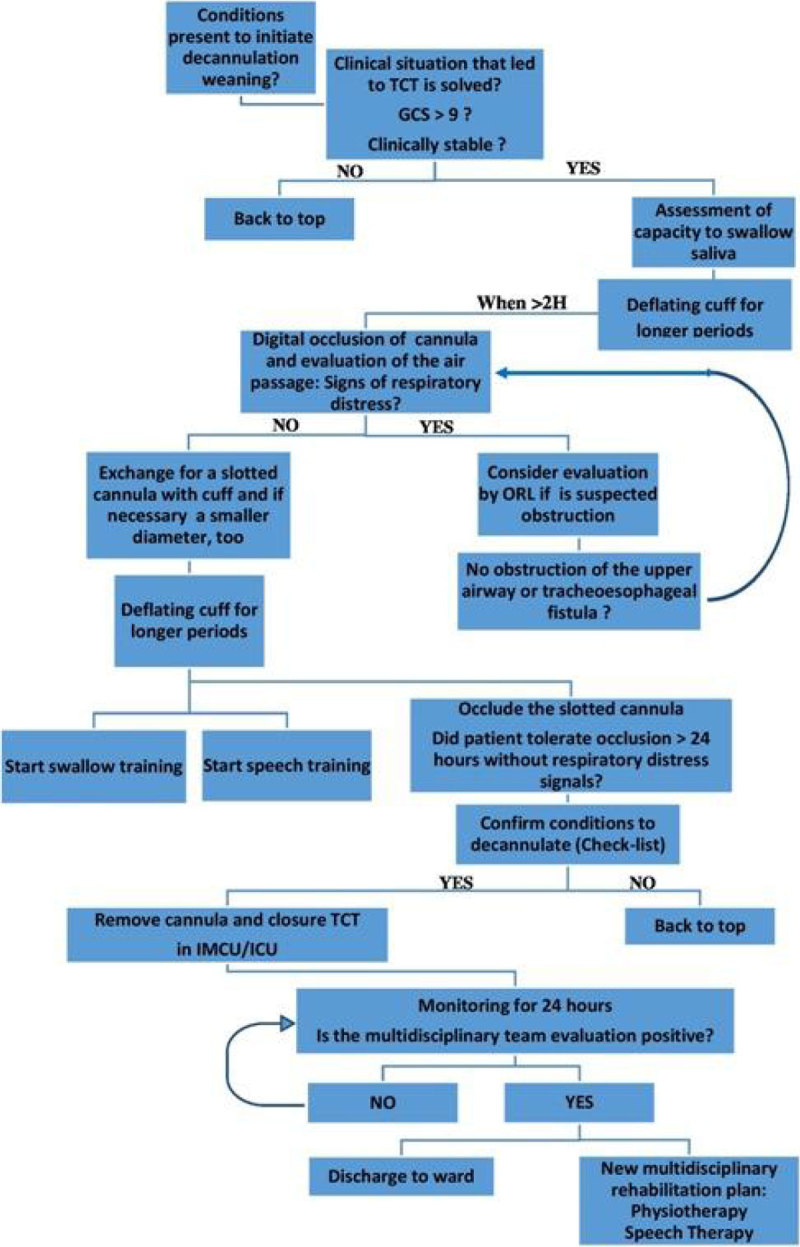
**Flowchart of decannulation protocol**.

From a total of 580 patients, 41 were submitted to TCT (7.1%); mean age was 66 years old; mean ICU length of stay was 25,7 days; reason for ICU admission was trauma in 45.5%, medical in 36.4%, urgent surgery in 15.2% and elective surgery in 3.0%; mean days of mechanical ventilation was 21,9 days; reason for TCT was, prolonged MV in 36.4%, Glasgow Coma Scale less than 9 in 54.5%; neuromuscular complication in 9.1%; mean days with TCT was 43.4 days; mean hospital stay was 78.8 days; percutaneous TCT was performed in 94% of patients; readmission rate was 24.2%; decannulation attempts failed: 0%. At 28 days: 66.7% of patients had TCT and 6.1% died. At 6 months: 24% of patients died and 12% remained in hospital (4% with TCT) and only 12% of patients were discharged to home. From the group of patients that were decannulated in ICU there were no deaths. The average length of the TCT in patients decannulated in the ICU was less than half compared to that obtained in patients decannulated in the wards (p < 0.05). The proposed protocol is presented in Figure 1.

## Conclusions

Understanding outcomes and trajectories of care in patients submitted to TCT was fundamental for the proposal of a decannulation protocol. Further studies are needed to evaluate the quality of life in TCT patients and provide further insights on this protocol optimization.

